# Delirium Tremens in Moderate Consumers of Alcohol

**Published:** 1899-08

**Authors:** Frank H. Pritchard

**Affiliations:** Monroeville, Ohio


### Delirium Tremens in Moderate Consumers of Alcohol.

BY FRANK H. PRITCHARD, M. D., MONROEVILLE, OHIO.

In the July number of *The Medical Times*, Dr. R. Elmergreen of Milwaukee, has reported four interesting cases of delirium tremens in moderate consumers of alcohol which are very instructive, and particularly with regard to their pathology. The writer has done us a service in calling our attention to this peculiar variety of delirium, for one may meet with them in practice, and they may be very embarrassing and treat one to unpleasant surprises. He seems to think that he has found a form of delirium tremens that stands apart from the usual one and which has something more or less mysterious about it. I must admit that the general text-books do not give much attention to this peculiar variety, and that it certainly is confusing. Osler, for example, does not offer much light on it.

My view of these cases is that they are usually uræmic and that through the moderate consumption of alcohol, and especially of beer, the kidneys are undergoing parenchymatous degeneration, possibly without any external signs or evidence of these lesions, and with the oncoming of a "congestive attack," as the English call it, or an acute congestion, or better said, an acute nephritis, in an already damaged kidney, the delirium is precipitated, the degenerated organs are unequal to the strain, and going from bad to worse, there is a general "smash-up" and with a rapidly rising temperature which, just before death, in the pre-agonic stage, reaches a grade at the top of the thermometer.

It will be noticed that in his first case there is a history of poor appetite, coated tongue and morning nausea, with gastric distress three months preceding his death, all symptoms certainly demanding an examination of the urine, and probably due to renal insufficiency and parenchymatous degeneration. His second case offered signs of an oncoming congestive attack from the shock of seeing his comrade mangled.

Moderate consumption of alcohol will sooner or later lead to sions or post-epileptic, but where it was noticed before the convulsions which set in. Here, in this group, the urine was dark, reddish brown, a little turbid, cloudy, and sometimes of a slimy consistence. The specific gravity was high, 1020-1035, the quantity greatly diminished, 100, 150 ccms. daily, or even complete anuria, for a day. There were casts of different kinds, renal epithelium, red and white blood corpuscles; these elements were most numerous at the height of the attack or immediately after. After the patient became quiet they disappeared.

The next group was of those with pronounced and well developed delirium without convulsions, who entered in the prodromal stage. There was albuminuria in them all, with renal epithelium and cylindroids, and in one fatal case a decided organic lesion was found post-mortem.

In another group which came under observation with delirium, without convulsions and during the delirious stage, in all there was albuminuria, in varying degrees, from a mere trace to goodly quantities. The state of the urine was less abnoraml than in those with convulsive phenomena, which agrees with the view that those cases with convulsions are the most serious.

The series of symptoms which are set forth as characteristic of latent nephritis have a striking similarity with those of the prodromal stage of delirium tremens. In both states there are both gastric and intestinal symptoms, cerebral and nervous phenomena, and, indeed, even nose-bleed is an accessory symptom in the not unusual hemorrhagic symptoms, which may be noted among the prodromes of Hertz's cases: hæmatemesis, epistaxis, bleeding from the gums and hæmaturia. Dieulafoy calls this state "petite urémie," and even without this hint one is led to look upon a condition which often shows itself with headache, and nearly always with nausea and vomiting or slight gastro-intestinal disturbances, together with renal troubles, as allied to uræmia. Then it is but a step to regard the whole delirium, together with the convulsions, as a sort of uræmia. Such a parallelism is not difficult to demonstrate. Convulsions, that typical uræmic symptom, appear in grave forms of uræmia as well as in severe cases of delirium tremens; the vomiting never continues into the stage where the severe cerebral degenerative changes in the kidneys, several writers to the contrary, notwithstanding, and that either directly or indirectly. Professor A. Villard of Marseilles—"Lecons sur l'Alcoolisme," p. 72, 1892—in speaking of the effect of this poison on the kidneys, says: "In whatever way that it be brought about, whether gradually or by repeated congestions, chronic nephritis is the usual fate of the steady drinker. Anatomically, you will find in general the small, granular and sclerosed kidney * * * and, indeed, the kidney of alcoholics is looked upon as the type of interstitial nephritis of arterial origin * * * with secondary mal-nutrition of the exciting cells and consequent degeneration. * * * In some cases you will discover instead of the small and contracted kidney a kidney but little or not at all reduced in volume, which on section offers brown or raspberry colored areas on a pale background—c'est le rein tacheté ou bigarré. In these kidneys the fundamental lesion is still a sclerosis, with areas of congestion or even of hemorrhage. Do not forget that the fatty degeneration of which I spoke as occurring in the liver may be met with in the kidney, concurring with cirrhosis—the small granulo-fatty kidney. At times the fatty degeneration will be found as existing alone, and particularly in beer drinkers. The kidney is then soft, of a white and lardaceous appearance; on section shining and greasy. The line of demarcation between the two portions is ill-defined. Thus you may meet with cases of moderate drinkers where uræmia suddenly sets in under various circumstances and suddenly puts an end to life, whose other organs seem to bear the fatal poisoning well."

So much for these statements. In 1898, in Nos. 8, 9 and 10 of the Danish medical journal, *Hospitalstidende*, Dr. Paul Hertz published a very interesting and instructive article on the "Pathogenesis of Delirium Tremens," which throws light on Dr. Elmergreen's cases. With admirable, patient and scientific thoroughness, which is characteristic of the Scandinavian physicians, he investigated for several months the cases of delirium tremens coming into the General Hospital in Copenhagen. He especially examined the urine of these patients before the delirium set in, when possible, as well as during and after. It was investigated immediately after entering, several times a day, microscopically and as to specific gravity, quantity, etc. He had 124 cases of undoubted delirium tremens, which he divides into groups. The gravest were those where there was a severe and uncomplicated attack, with convulsions—nine cases—all with albuminuria, which was noted in the prodromal stage, in goodly quantities and which was not a consequence of the convulsymptoms appear, as in delirium tremens. The delirium of uræmia is well known. Toulouse has made it the object of a thorough study—"Les Troubles Méntaux de L'Urémie," *Gazette des Hospitaux.*

No. 70, 1894—and he characterizes it as an acute hallucinatory delirium. Delirium tremens is exactly the same form of psychosis. Indeed, one French writer, Lecorché, asserts that uræmic delirium may be still and quiet or violent, and easily confounded with delirium from other causes, as for example, that of alcoholism. That uræmic delirium more frequently is joined with convulsive than with comatose forms of uræmia also points to a relation with delirium tremens.

The temperature in delirium tremens has been difficult to understand, but possibly it is more easily comprehended if one remember that it depends upon similar causes to that in uræmia. Hughes and Carter—"A Clinical and Experimental Study of Uræmia," *American Journal of the Medical Sciences,* Vol. 108, 1894—claim that the temperature in uræmia is no constant nor pronounced symptom. It may be either above, below or at the normal. It is more frequently observed above in parenchymatous nephritis. Stengel—"Fever in Nephritis," *American Journal of the Medical Sciences,* Vol. 110, 1895—mentions that the temperature may rise with coma or delirium or convulsions. Dethlefsen has found rises of temperature after convulsions in forty-two per cent. of his cases.

Finally, Hughes and Carter have said that uræmia may set in without the urine being albuminous and yet the necropsy reveal a well developed renal disease. This must be comparatively rare, though I have noted the albumen in some renal affections to vanish at times, and to be plentiful at others. Therefore, the following conclusion may be drawn: if insufficiency of the renal functions during that state which is called uræmia, is able to produce hallucinatory confusion, therefore it is not illogical to assume that the hallucinatory confusion which is called delirium tremens and which always has other symptoms in common with uræmia, is dependent upon an "insufficiency of the renal functions" when there are always signs of disturbed kidney function associated with it.

Hertz concludes his lengthy and very instructive article as follows: As a result of my investigations it may be concluded that:

1. A renal disturbance is a constant accompaniment of uncomplicated delirium tremens.

2. That the relation of time between the renal disturbance and the appearance of the delirium shows that the renal disease is primary and the delirium secondary.

3. That the anatomical base of the renal disturbance is an acute nephritis, which, as a rule, probably develops without any preceding chronic nephritis.

4. That the course of the renal disturbance follows so closely, step by step, with the delirium that there is good ground for assuming that there is a genetic connection between the two phenomena.

5. That there are so many similar points in the two states which are notoriously brought about by an insufficiency of the renal functions (uræmia), and delirium tremens, that there is reason to assume that *the delirium is an acute auto intoxication-psychosis as a consequence of the insufficient kidney function, which is due to the acute nephritis.*

6. That the peculiar form that this psychosis takes on is dependent upon its developing in chronic alcoholics.

Finally, 7. That there is a probability that delirium tremens in pneumonia is dependent not on the pneumo-toxines directly, but the always present renal lesion.

I have examined the urine of quite a number of beer and whiskey drinkers, and I have found that even when they were in fair condition yet their kidneys were not nomal, for the urine would be thin, with a slight trace of albumen, a few renal epithelia and an occasional cast, if they were a little under the normal. If they would take cold the quantity of albumen would increase, hyaline casts be thrown off, and symptoms of renal inadequacy set in. On leaving off the habit the urine would quickly or slowly return to the normal, according to the amount of renal mischief already done; of course, if decided alterations had already occurred, then much less restoration would follow. In short, I doubt whether the kidneys in cases of delirium tremens were so normal before the attack, and whether the renal insufficiency had developed on such virgin soil as Hertz would assume. He had no means of examining the urine of these patients weeks before they entered. His evidence is somewhat contradictory, for on page 239 he asserts that in about one-third of the cases he had found pre-existing symptoms of chronic nephritis, in others stethoscopic and physical signs of a hypertrophy of the heart. He assumes that the delirium develops not with a background of a chronic renal disease, but as an actual acute process. I would not attempt to dispute him, but I have gained the impression that alcoholics have kidneys of lesser resistance. Dr. Elmergreen tells me nothing about the urine in his cases. Possibly he might have had more light had he examined the urine carefully.—*Medical Times.*

